# 

**Published:** 1923-07

**Authors:** 


### PUBLIC HEALTH IN PARLIAMENT.

The following are selected as being of general interest from answers recently given by the Minister of Health to questions in Parliament.

### NATIONAL HEALTH INSURANCE.

Panel System.—The revision of the terms of service of insurance practitioners with a view to securing a more efficient medical service under the Insurance Acts has been fully examined in consultation with the Insurance Consultative Council and the Insurance Acts Committee of the British Medical Association, and I do not consider that any useful purpose would be served by such an enquiry [into the working of the panel system] as the Hon. Members suggest.

Panel Doctors (Records).—I cannot accept the suggestion implied in the Hon. Member's question that the keeping of the prescribed simple form of record need interfere in any way with the proper examination of patients, and I am not prepared to assent to the abolition of the record card.

Agricultural Workers (Benefits).—As agricultural workers are distributed among many thousand Approved Societies and branches which are not, in general, organised on an occupational basis, it is impossible to give figures as to the annual value of benefits received by such workers throughout the country. An analysis of the figures of certain societies whose membership consists largely of agricultural workers shows a claims experience from 5 to 10 per cent. more favourable than that of societies generally, with the result that such societies have been placed in a position to provide their members with exceptionally favourable additional benefits.

Approved Societies (Finance).—Out of the disposable surpluses of Approved Societies in England, following the first valuation, the aggregate amounts set aside for provision of the benefits in question during the five years covered by the schemes were as follows :—

| | |
|---|---|
| Dental Treatment ... ... ... ... | £495,000 |
| Optical Treatment ... ... ... ... | 106,000 |
| Hospitals and Convalescent Homes ... | 979,000 |
| Medical and Surgical Appliances ... ... | 164,000 |
| Nursing ... ... ... ... ... ... | 207,000 |

### HOUSING.

Local Authorities' Schemes.—I appreciate that in some districts it may not be practicable to secure the immediate erection of houses by private enterprise, and I should in such cases be prepared to consider an application from the local authority to erect houses themselves. It is desirable, however, that in such cases the proposals should be limited in the first instance to such number of houses as can be commenced and finished quickly, and that during the construction of these houses the local authority should carefully examine what steps can best be taken to encourage the erection by other agencies of the houses required.

### TUBERCULOSIS.

School Children.—By the President of the Board of Education: The relevant statistics of the Board divide children into two categories, those ascertained at medical inspection to be suffering from pulmonary tuberculosis, and those ascertained to be suffering from crippling due to tuberculosis. According to the latest returns furnished by local education authorities, the number of children in the first category is, approximately, 20,000, of whom about 2,000 are in special schools, 11,000 are in public elementary schools, and 7,000 are attending institutions not certified by the Board or are not at school. In the second category, there are 13,000 children, of whom 1,700 are in special schools, 6,500 are in public elementary schools, and 4,800 are attending institutions not certified by the Board or are not at school.

### CANCER.

Grants for Research.—Financial assistance is at present given from public funds to cancer research through the Medical Research Council, who, in the last financial year, expended a sum of approximately £5,000 for this purpose in addition to further large sums assigned to the support of other investigations not specifically directed toward the problem of cancer, but which may, nevertheless, provide the desired clues. A special departmental committee was appointed in January last to survey the whole question.

### RECENT APPOINTMENTS.

### Medical Officers, etc.

Bain, C. W. C., M.C., M.R.C.S., L.R.C.P., Resident House Physician, St. Thomas's Hospital.

Churcher, J. C., M.R.C.S., L.R.C.P., Resident House Surgeon, St. Thomas's Hospital.

Cohen, S. M., M.D., M.R.C.S. ; certifying surgeon under the Factories and Workshops Act for the Rhondda district.

Drew, J. G., M.R.C.S., L.R.C.P., Resident House Physician, St. Thomas's Hospital.

Dunscombe, C., M.R.C.S., L.R.C.P. (London), D.P.H. Resident Medical Officer, Brighton Isolation Hospital.

Feiling, Anthony, M.D., M.R.C.S., L.R.C.P. ; Assistant Physician, St. George's Hospital.

Gainsborough, Hugh, M.R.C.S., Resident Assistant Physician, St. George's Hospital; Assistant Physician, St. George's Hospital.

Gaydon, F. A., M.R.C.S., L.R.C.P., Resident House Physician, St. Thomas's Hospital.

Gibson, J. M., M.D., D.P.H., M.O.H. and School Medical Officer, Radcliffe Urban District.

Hart, Bernard, Radiographer, Doncaster Infirmary.

Leys, D. G., B.A., M.B., Resident House Physician, St. Thomas's Hospital.

Lough, Muriel Jessie, M.R.C.S., L.R.C.P., M.O., Newark Street Municipal Infant Welfare Centre, Stepney: Woman Assistant M.O. Urban District Council of Barking Town. (London School of Medicine for Women and St. Mary's Hospital, Paddington.)

Maclean, N. A., M.B., Ch.B., House Surgeon, Addenbrooke's Hospital, Cambridge.

Macpherson, N. S., M.R.C.S., L.R.C.P., Resident House Surgeon, St. Thomas's Hospital.

Mason, H., M.B., D.P.H., Chief Assistant School M.O.' North Riding ; County M.O.H., North Riding. (£1,00 .)

Nichol, R. W., M.R.C.S., L.R.C.P., Resident House Surgeon, St. Thomas's Hospital.

Oliver, C. P., M.R.C.S., L.R.C.P., Resident House Physician, St. Thomas's Hospital.

Patrick, C. V., M.R.C.S., L.R.C.P., Resident House Surgeon, St. Thomas's Hospital.

Potts, Thomas Norman Vickers, M.B., B S., Assistant M.O.H., Blackburn ; Assistant M.O.H., Newcastle-on-Tyne. (Durham University.) £750-£900.

Proctor, Ruth E., M.A.M.B., Ch.B., D.P.H., Assistant M.O., Bromley Town Council; Assistant M.O., County Council of Middlesex (St. Andrew's University, D.P.H., London University).

Roe, George Charles Frederick, L.R.C.P., L.R.C.S., D.P.H., Irel., Assistant M.O., St. Patrick's Hospital, Dublin ; Assistant M.O.H., City of Wakefield. (Meath Hospital and Dublin County Infirmary and Schools of Surgery of the Royal College of Surgeons).

Smith, E. Bellingham, A.M.D., Assistant Physician, St. George's Hospital ; Physician, St. George's Hospital.

Towers, J. A., M.R.C.S., L.R.C.P., Assistant Physician, St. George's Hospital ; Physician, St. George's Hospital.

### Public Health and Hospital Appointments.

Burn, Mary Louisa, Staff Nurse, Tynemouth Union Hospital ; Health Visitor, Sunderland. (Harton Hospital, South Shields.)

Howarth, Ada Alice, C.M.B., Health Visitor and School Nurse, Hyde. (Chesterfield Union Infirmary.)

Shuttleworth, Annie, C.M.B., Health Visitor and School Nurse, Hyde. (Leeds Township Infirmary.)

Spencer, Ethel Frances, office sister and locum assistant matron, St. Thomas's Hospital ; Matron, Royal Sussex County Hospital. (St. Thomas's Hospital.)

